# De novo assembly and single-molecule study of kinetochore-microtubule interactions

**DOI:** 10.3389/fcell.2025.1724413

**Published:** 2026-01-12

**Authors:** Joshua D. Larson, Lillian R. Worst, Charles L. Asbury

**Affiliations:** 1 Department of Neurobiology & Biophysics, University of Washington, Seattle, WA, United States; 2 Department of Biochemistry, University of Washington, Seattle, WA, United States

**Keywords:** cell division, mitosis, mitotic spindle, microtubule polarity, plus end preference, TIRF microscopy

## Abstract

Kinetochores are essential molecular machines composed of dozens of protein subcomplexes that assemble onto specialized centromeric nucleosomes during every cell cycle prior to mitosis. During mitosis, the assembled kinetochores are responsible for maintaining load-bearing attachments to dynamic spindle microtubules, and for harnessing the forces generated by attached microtubules to organize and separate sister chromatids. Recent work shows that kinetochores can be reconstituted by assembling them *in vitro* onto centromeric DNAs in yeast whole cell lysates. By tethering individual centromeric DNAs to the surface of a coverslip, the assembly process and the microtubule-attachment activity of the assembled kinetochores can be studied at the single-molecule level. Kinetochores reconstituted in this manner are able to capture taxol-stabilized microtubules, with a strong intrinsic preference specifically for capturing microtubule plus ends. Super-resolution tracking further shows that the architecture of the assembled kinetochores changes in a microtubule polarity-dependent manner under external load. We anticipate that extensions of these approaches will uncover the molecular basis of the kinetochore’s plus end-preference and, ultimately, will reveal how tension affects the arrangement of core subcomplexes and transient regulatory factors. Here we detail how to study individual kinetochores assembled from yeast whole cell lysate using single-molecule total internal reflection fluorescence microscopy.

## 1 Introduction

Many of life’s essential cellular processes are carried out by megadalton multi-component molecular machines. *In vivo* studies of these assemblies, while powerful, are limited in their ability to interrogate key biochemical and biophysical features, in part because the assemblies often have ancillary functions in addition to their primary purpose, which can be difficult to deconvolute. *In vitro* studies, in which part or all of a cellular complex can be examined in isolation, provide a more controlled environment and allow for a more surgical dissection of the various forms and functions of complicated molecular machines. The ability to reconstitute assemblies for *in vitro* studies is crucial for understanding how they function and how defects contribute to human disease. Complexes with large numbers of molecular components, however, can make reconstitutions based on recombinantly purified proteins inefficient or outright prohibitive. In early attempts to reconstitute ribosomes, for example, assembly would halt at distinct intermediates or off-pathway products that prevented the formation of active particles (Held et al., 1973). Bypassing these bottlenecks typically required exposure to high heat, high salt concentrations, or eventual discovery of additional co-factors that allowed assembly to proceed (Tamaru et al., 2018). While assembling ribosomes using recombinantly purified subunits is possible today, reconstitutions of other large, essential molecular complexes, such as those involved in gene expression or cell division, have yet to be fully achieved in this manner. An alternative approach is to assemble them in whole cell lysates. Assembly in cell lysates offers several advantages over recombinant approaches. First, it circumvents the need to purify recombinant proteins. Second, regulatory elements or transient subcomplexes required for assembly or function are often present and active in cell lysates, including factors yet to be identified. Finally, similar to studies using purified recombinant proteins, *in vitro* studies in whole cell lysates are often amenable to biochemical and biophysical methodologies that are prohibitive for *in vivo* studies.

One macromolecular machine that has yet to be fully reconstituted is the kinetochore. Kinetochores are essential for accurate chromosome segregation during cell division. They are composed of dozens of protein subcomplexes that assemble on specialized centromeric nucleosomes. Kinetochores are responsible for maintaining load-bearing attachments to dynamic spindle microtubules and harnessing the forces generated by dynamic microtubules to segregate sister chromatids during mitosis (Biggins, 2013; Hinshaw and Harrison, 2018; Musacchio and Desai, 2017). Kinetochores also ensure accurate segregation by identifying and eliminating erroneous microtubule attachments and delaying entry into anaphase until all erroneous attachments have been corrected (Li et al., 2024; McAinsh and Kops, 2023). These two regulatory functions depend on subcomplexes and cofactors whose association with the kinetochore and/or activation varies depending on the status of microtubule attachment. Many partial kinetochore assemblies have been reconstituted from individually purified subcomplexes. Outer kinetochore subcomplexes that harbor microtubule-binding elements have been reconstituted for structural and biophysical analysis (Asbury et al., 2006; Ciferri et al., 2008; Dimitrova et al., 2016; Muir et al., 2023; Powers et al., 2009; Yatskevich et al., 2024). In separate structural studies, inner kinetochore subcomplexes bound to the centromeric nucleosome have been reconstituted, but required the use of non-native centromeric DNA or artificial protein scaffolding for stability (Dendooven et al., 2023; Guan et al., 2021). The studies closest to reconstituting the activities of complete kinetochores have isolated native kinetochore particles from *Saccharomyces cerevisiae* (budding yeast) by immunoprecipitation (Akiyoshi et al., 2010; Barrero et al., 2024; Miller et al., 2016) or assembled minimal chains of recombinantly purified inner, middle, and outer kinetochore subcomplexes (Hamilton et al., 2020). Both these types of kinetochore assemblies were competent for microtubule binding and amenable to biophysical interrogation. However, neither approach fully recapitulated complete kinetochores built on DNA-wrapped centromeric nucleosomes, and many of the transiently associated regulatory subunits required for error correction and mitotic regulation were absent in these studies.

More recent studies have reconstituted kinetochores by assembling them *de novo* in budding yeast whole cell lysates. Initially, yeast centromeric DNA was conjugated to magnetic beads, which were then incubated in the lysates (Lang et al., 2018). Examination of these particles by Western blotting and mass spectrometry indicated that the assemblies contained all the constitutively associated kinetochore subunits, all three of the microtubule binding elements, the centromeric nucleosome, and the chromosomal passenger complex, which is key for correcting erroneous microtubule attachments. By conjugating the centromeric DNAs to the surface of a coverslip, these *de novo* assembled kinetochore particles become amenable to single molecule biophysical studies (Larson et al., 2025; Popchock et al., 2025).

**FIGURE 1:**
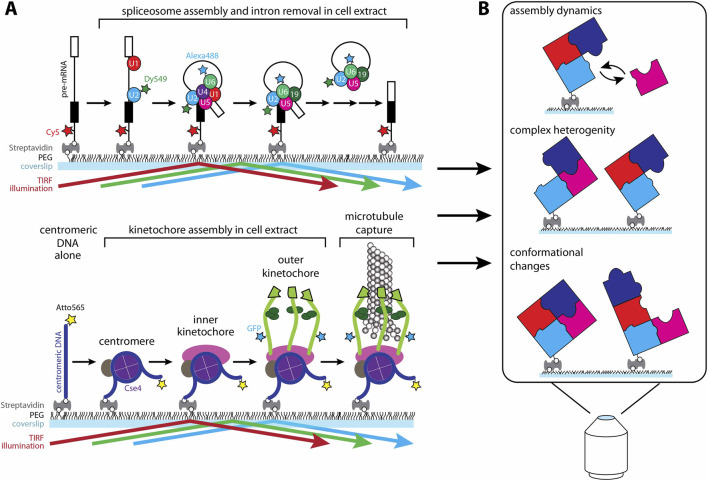
Using single molecule total internal fluorescence reflection microscopy to study large macromolecular complexes assembled in whole cell lysate. **(A)** Cartoon depictions of two examples of single molecule experiments to study the biochemical and biophysical properties of essential macromolecular complexes in whole cell lysate. (*Top*) Single fluorescently labeled pre-mRNAs are conjugated to the surface of a passivated coverslip. Labeled spliceosomes assemble on the pre-mRNA and excise the intron. (*Bottom*) Single fluorescently labeled centromeric DNAs are conjugated to the surface of a coverslip. Labeled kinetochores assemble on the centromeric DNA and capture microtubules. **(B)** Examples of the types of biochemical and biophysical information that can be analyzed using macromolecular complexes assembled in whole cell lysate.

Cellular lysates are profoundly heterogenous mixtures in which a majority of molecular components are not associated with the complex of interest. Additionally, many of the macromolecular complexes of interest are both dynamic and heterogenous themselves, obfuscating interpretation in bulk biochemical assays. One way to overcome the limitations of lysates is to utilize single-molecule methods to ‘highlight’ complexes of interest within the slurry of intercellular components. This method was pioneered to study spliceosome assembly (Crawford et al., 2008; Hoskins et al., 2011). Yeast lysates containing fluorescently labeled spliceosome subcomplexes were incubated with fluorescent pre-mRNAs conjugated to the surface of a coverslip, and total internal reflection microscopy (TIRFM) was used to observe both spliceosome assembly and intron excision, two processes that had not previously been observed in real-time (Figure 1A, *top*). Similarly, early kinetochore assembly can be observed by TIRFM when fluorescently tagged yeast centromeric DNA molecules are conjugated to a coverslip and incubated with yeast whole cell lysate containing fluorescently tagged kinetochore components (Figure 1A, *bottom*) (Hu et al., 2025; Larson et al., 2025; Popchock et al., 2025). Because kinetochores are stable once assembled in lysate, the lysate can be washed away, leaving the individual kinetochore assemblies isolated on the coverslip surface, for observation and study without the confounding aspects of a protein-dense slurry. Kinetochores reconstituted in this manner can capture taxol-stabilized microtubules, with a strong intrinsic preference specifically for capturing their plus ends (Larson et al., 2025). Super-resolution tracking further shows that the architecture of these assembled kinetochores changes in a microtubule polarity-dependent manner under external load (Larson et al., 2025). In the future, we anticipate that extensions of these approaches will uncover the molecular basis of the kinetochore’s plus end-preference and, ultimately, will reveal how tension affects the arrangement of core subcomplexes and transient regulatory factors. Here we detail how to prepare and study individual kinetochores assembled from yeast whole cell lysate with single-molecule TIRFM.

## 2 Overview

To study the activity of assembled kinetochores using single-molecule TIRFM, several preparatory steps must be taken before the day of the assembly assay. Here we describe each of the steps needed to prepare for and execute kinetochore assembly and microtubule capture. We begin with protocols for making yeast whole cell lysate, fluorescent centromeric DNAs, fluorescent microtubules, and passivated flow chambers. Each section begins with a summary of the purpose of the protocol and noteworthy things to consider while following the protocol. Next is a step-by-step set of instructions. Each section includes footnotes with additional advice or details pertaining to one or more of the steps that may help provide clarity. Several of the sections are branched and describe two or more similar methods that can be interchanged based on the goal of the experiment. (E.g., does the experiment require the use of polarity-marked microtubules or are uniformly labeled fluorescent microtubules sufficient?) The manuscript concludes with a step-by-step guide for completing three distinct but related experiments, the assembly of individual kinetochores in yeast lysate on the surface of a coverslip, the capture of single microtubules by assembled kinetochores, and the ‘flip-flop’ assay that we use to measure intra-kinetochore distances using oscillating flow. Image acquisition and data analysis procedures are detailed.

We note that careful consideration is needed when selecting fluorescent labels for tagging each molecular component of interest, so that spectral separation and signal-to-noise are optimized for your TIRFM’s optical configuration. Here we describe kinetochore assembly and microtubule capture assays with a labeling scheme specifically matched to our TIRFM instrument (Deng and Asbury, 2017). Our instrument uses 488-nm, 561-nm, and 647-nm diode lasers to excite GFP-labeled kinetochores, Atto555-labeled centromeric DNAs, and Alexafluor647-labeled microtubules, respectively. The emission from the three labels is separated and directed to three Andor iXon987+ EMCCDs by two long-pass filters (560LP and 640LP).

### 2.1 Preparation of whole cell lysates

Yeast whole cell lysate will need to be prepared in advance of kinetochore assembly and microtubule capture assays. Lysate preparation begins with growing the selected yeast strain, typically with one GFP-tagged kinetochore component, to the appropriate optical density and volume, which affects the final protein concentration and volume of lysate. Yeast can be grown asynchronously or, optionally, add benomyl to the culture to ensure most cells are in metaphase at the time of harvest. To produce highly active lysates, harvesting is best done at a high optical density while the yeast are in log phase. Lysis of yeast cells is accomplished with a SPEX SamplePrep freezer mill, and the lysate is subsequently clarified with centrifugation. For the protocol described here, a standard tabletop centrifuge is all that is needed. Other studies include additional clarification steps with an ultracentrifuge (Hoskins et al., 2011), which can help reduce the fluorescence background of the lysate during imaging without reducing activity. Careful attention should be paid to the final protein concentration. The optimal concentration for the assays described here is between 40 and 80 mg/mL. The concentration of the lysate can be adjusted by changing the volume of buffer used to resuspend the cells after harvesting but before lysis with the freezer mill. For best results, it is important to produce lysates with consistent protein concentrations.

1. Grow 2L of cells to an OD600 of 4–5 in YPED.
2. Centrifuge the cells for 5 min at 2,500 x g, 4 °C.
3. Resuspend the cell pellets in a total volume of 80 mL of MilliQ water with 0.2 mM PMSF.
4. Divide the cell suspension evenly into four 50 mL falcon tubes and centrifuge for 3 min at 2,500 x g, 4 °C.
5. Decant the supernatant and add 10 mL of lysis buffer (recipe below) to each falcon tube.^1^
  25 mM HEPES pH 7.6.
  6 mM Magnesium Acetate.
  0.1 mM EDTA.
  0.5 mM EGTA.
  15% Glycerol.
  0.1% NP-40.
  175 mM Potassium Glutamate.
  2 mM DTT.
  0.2 mM PMSF.
  10 μg/mL Leupeptin.
  10 μg/mL Pepstatin.
  10 μg/mL Chymostatin.
6. Centrifuge the cells for 3 min at 2,500 × g, 4 °C.
7. Decant the supernatant and resuspend the cells with an appropriate volume of lysis buffer so that the lysate will have the desired protein concentration.^2^
8. Freeze the cell slurry by slowly dripping it into liquid nitrogen, forming frozen cell pellets.^3^
9. Lyse the cell pellets in a freezer mill (10 rounds at 10 cycles per second with 2 min of lysing and 2 min of cooling.
10. Thaw the lysate powder on ice and transfer 1.5 mL eppendorf tubes once thawed.
11. Centrifuge for 30 min at 17,000 × g, 4 °C.
12. Pipet off the supernatant into a 15 mL falcon tube and place on ice.
13. Make single-use aliquots and snap freeze in liquid nitrogen.^4^

### 2.2 Preparation of fluorescently labeled centromeric DNAs

208-bp Atto565-labeled wild type and mutant centromeric DNAs are generated by PCR from plasmids pSB963 and pSB972, respectively, which are both based on the centromeric DNA sequence from *S. cerevisiae* chromosome III (CEN3). For non-functional negative control mutant centromeric DNAs, a 3-bp substitution can be made in the CDEIII region that blocks kinetochore assembly *in vivo* (Lechner and Carbon, 1991; Sorger et al., 1994; Sorger et al., 1995) and *in vitro* (Lang et al., 2018). The forward primer contains a 5′biotin for specific attachment to the coverslip and the reverse primer is labeled with Atto565. Both primers can be custom-synthesized, including the biotin and Atto565 labels, by Integrated DNA Technologies.

#### 2.2.1 PCR amplify using NEB Taq polymerase eight 50 µL reactions

Per 50 µL reaction, combine:

1. 5 μL 10X ThermoPol Reaction Buffer.
2. 1 μL 10 mM dNTPs.
3. 1 μL 10 µM forward primer.
4. 1 μL 10 µM reverse primer.
5. 1 μL 1:100 template DNA (∼1 ng or less of plasmid).
6. 0.25 µL NEB Taq polymerase.
7. 40.75 µL MilliQ water to 50 mL.

Thermal cycling protocol:

1. 95 °C, 30 s.
2. 95 °C, 15–30 s.
3. 45 °C–68 °C, 30–60 s.
4. 68 °C, 1 min per kb.
5. Repeat steps 2 through 4, 29x.
6. 68 °C, 5 min.

Clean up the DNA using a PCR Purification Kit. (Note: the Promega Wizard Kit has high capacity.) Use one column for every eight 50 µL reactions (1 PCR strip). Elute in 50 µL MilliQ water per column.

### 2.3 Preparation of fluorescent microtubules

Fluorescent microtubules should use a different-colored dye than either the labeled kinetochore components in the yeast extract or the DNA. We assemble the microtubules using tubulin labeled with HiLyte 647 (Cytoskeleton, Inc.). If the proportion of fluorescent tubulin is too high, it can interfere with microtubule growth. Uniformly fluorescent microtubules suffice when it is not necessary to know the polarity of the microtubules. Polarity-marked microtubules with a dim plus end and bright minus end make it possible to determine which end of the microtubule was captured by the kinetochore (Roostalu et al., 2011), but these are more difficult to make and have a shorter shelf-life than uniformly fluorescent microtubules. The tubulin mixes for assembling polarity-marked microtubules can be made ahead and stored indefinitely at −80 °C. Microtubules stabilized by taxol can be stored in the dark at room temperature for up to a week before use. Because the concentration of microtubules can be highly variable, it is good practice to check their density before diluting them for the assay.

#### 2.3.1 Uniformly fluorescent microtubules

1. Make 125 µL of microtubule polymerization buffer (MBP) by combining:
  85.8 µL of MilliQ water.
  24 μL of 5× BRB80 (400 mM PIPES pH 6.8, 5 mM MgCl_2_, 2.5 mM EGTA).
  7 μL of DMSO.
  2 μL 250 mM MgCl_2_.
  1.2 µL 100 mM GTP.
2. Resuspend HiLyte 647 tubulin with unlabeled bovine tubulin so that the HiLyte 647 tubulin is ∼6% of the total tubulin.
3. Combine 12 µL of tubulin mix with 48 µL of MPB and incubate at 37 °C for 1 h.
4. Add 140 µL of warm (37 °C) 1x BRB80 plus 10 µM taxol to the polymerized microtubules.
5. Spin at 19,000 x g for 10 min.
6. Gently wash the microtubule pellet with 1xBRB80 plus taxol a few times.
7. Gently resuspend the pellet in 150 µL of 1xBRB80 plus taxol.
8. Microtubules can be stored at room temperature in the dark for up to 2 weeks.

#### 2.3.2 Polarity-marked fluorescent microtubules

Two different tubulin mixes with different ratios of fluorescent tubulin to unlabeled tubulin will need to be prepared in order to make polarity-marked microtubules. A ‘bright-tubulin’ mix, with a higher ratio of fluorescent to unlabeled tubulin is used to polymerize a bright seed. A subsequent polymerization step with a ‘dim-tubulin’ mix containing a lower amount of fluorescent tubulin will create dim extensions from the bright seed. Since microtubules polymerize faster from the plus end, we can differentiate between the plus and minus end based on the length of the dim extensions. Adding N-ethylmaleimide (NEM) treated tubulin will further inhibit growth at the minus end, making plus end identification easier. The optimal ratios of fluorescent and unlabeled tubulin for the tubulin mixes can vary depending on specifications of the TIRFM instrument. The goal is to produce microtubules with dim extensions that are clearly visible and distinct from the bright seeds. Special care is needed to warm the dim tubulin mix quickly before addition of the polymerized bright seeds.

It is imperative to carefully control the temperatures of the various mixtures throughout the process of making microtubules to prevent depolymerization which would remove the polarity mark. After removing the bright seed mix from the freezer, it should be kept at 37 °C until the addition of taxol makes the microtubules more stable. This includes when adding the dim seed mix, which needs to be warmed to 37 °C immediately before addition, so that it does not cool the polymerized bright mix, but only briefly so that it does not have time to begin polymerizing as uniformly dim microtubules. The tubulin mixes should also be kept cold when they are being made, to prevent premature polymerization. Bright seeds and dim extension lengths can be varied by changing the two incubation times. 30–45 min for the first bright incubation followed by 45–60 min for the second incubation tends to work well in our hands. If the microtubules are too long or too short, it can help to vary the incubation times.

##### 2.3.2.1 Dim unpolymerized NEM-treated tubulin mix

1. Make 50 µL NEM treated tubulin mix by combining the following:
  40 μM (final concentration against water) unlabeled bovine tubulin (volume will vary).
  2.5 µL 10 mM GMPcPP (final concentration 0.5 mM).
  2 μL 25 mM NEM (final concentration 1 mM).
  10 μL 5x BRB80.
  Add MilliQ water to a final volume of 48 µL.
2. Incubate on ice for 10 min.
3. Quench with 2 µL of 200 mM β-mercaptoethanol.
4. In a fresh tube, mix:
  Total final tubulin concentration of 20 µM.
    5% (1 µM) HiLyte 647 tubulin.
    45% (9 µM) NEM treated tubulin from step 1.
    50% (10 µM) unlabeled bovine tubulin.
    20 μL 10 mM DTT (final concentration 1 mM).
    10 μL 10 mM GMPcPP (final concentration 1 mM).
    40 μL 5x BRB80.
  Bring the volume to 200 µL with deionized water.
5. Incubate the mixture on ice for 5–10 min.
6. Spin the tubulin mix at 300,000 × g, 4 °C for 30 min to remove any polymerized tubulin or possible contaminates and make 30 µL aliquots of supernatant. Snap freeze in liquid nitrogen and store at −80 °C until ready to assemble microtubules.

##### 2.3.2.2 Bright unpolymerized tubulin mix

1. In a fresh tube, mix:
  Total tubulin concentration of 20 µM made up of:
    30% (6 µM) HiLyte 647 tubulin.
    70% (14 µM) unlabeled bovine tubulin.
    3.5 µL 10 mM DTT (final concentration 1 mM).
    3.5 µL 10 mM GMPcPP (final concentration 1 mM).
    7 μL 5x BRB80.
  Bring the total volume to 35 µL with deionized water.
2. Incubate on ice for 5–10 min.
3. Spin the tubulin mix in a TLA100 rotor at 300,000 × g, 4 °C for 30 min to remove any polymerized tubulin or possible contamination and make 5 µL aliquots of supernatant. Snap freeze in liquid nitrogen and store at −80 °C until ready to assemble microtubules.

##### 2.3.2.3 Assembling the polarity-marked microtubules using bright and dim mixes

1. Thaw one aliquot of bright tubulin mix at 37 °C. Add 45 µL of warm 1 mM DTT in 1x BRB80, mix gently, and incubate at 37 °C for 30–45 min.
2. Approximately 10 min before the end of the bright tubulin incubation, retrieve an aliquot of dim tubulin mix and thaw it on ice. When bright tubulin incubation is done, add 30 µL of dim tubulin mix to 140 µL of cold 1 mM DTT in 1x BRB80.
3. Warm this mixture for 30 s at 37 °C, then immediately add 50 µL of the polymerized bright tubulin.
4. Incubate for 45 min to 1 h at 37 °C.
5. After incubation, stop polymerization and add taxol to a final concentration of 10 μM, mix very gently.
6. Centrifuge the mixture at 19,000 × g, 37 °C for 5 min.
7. Carefully remove and discard supernatant.
8. Gently wash the pellet once with 50 µL of 10 µM taxol in 1xBRB80.
9. Resuspend the pellet in 150 µL of 10 µM taxol in 1xBRB80.
10. Check the microtubules by adding about 8 µL to a plasma cleaned microscope slide with a shallow tape channel and viewing on the TIRF. Microtubules should be dense, and make sure that the polarity mark is easily distinguishable in at least 70% in a field of view.
11. Polarity-marked microtubules can be stored for a few days at room temperature in the dark, but should be used within a week, and can be sensitive to cooler room temperatures.

### 2.4 Preparation of flow-channels

In order to prepare flow-channels for the kinetochore assembly assay described below in part 2.5, we start by cleaning the slides and coverslips using detergent, etching them with potassium hydroxide, and then activating them with Vectabond (aminosilicate/APTES). Then we create channels by adhering the coverslips to slides with thick double-sided tape. The thick tape gives height in the channel to facilitate the flowing of viscous solutions through the channel. We then passivate the glass surfaces inside each channel overnight with a mixture of polyethylene glycol (PEG) and biotin-PEG. This passivation prevents non-specific binding, while allowing specific binding of the biotinylated DNA to the surface later.

#### 2.4.1 Cleaning and activating slides and coverslips

1. Gather a slide mailer, two microscope slides,^5^ and three coverslips.^6^ Use a plasma cleaner to clean the surfaces of the slides and coverslips for about 10 min, then transfer them into the slide mailer.
2. Fill the slide mailer with 2% micro90 detergent until slides are fully submerged (∼25 mL).
3. Sonicate the slide mailer for 30 min to 1 h.
4. Pour off the micro90 and rinse five times with milliQ water. Completely replace the volume each rinse.
5. Add anhydrous alcohol until slides are submerged, return to sonicator, and sonicate for 30 min to 1 h.
6. Rinse five times with water.
7. Add 1 M KOH, sonicate for 1 h.
8. Rinse five times with water.
9. Add water, sonicate for 30 min.
10. Dry the slides with nitrogen and add to a new slide mailer.
11. In a falcon tube, combine 20–25 mL acetone with 300 µL of Vectabond (aminosilicate/APTES).
12. Cover the cleaned slides in the Vectabond solution, and incubate in the dark for at least 10 min.
13. Remove the Vectabond, and wash seven times with anhydrous alcohol.
14. Dry with nitrogen and place in a new (third) mailer to avoid contaminating the slides.^7^

#### 2.4.2 Preparation of simple double-open-ended flow-channels

**FIGURE 2:**
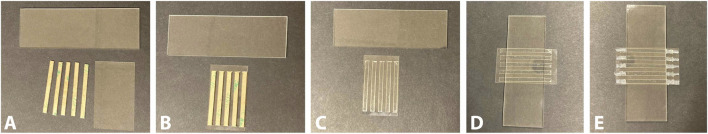
Constructing simple, double-open-ended flow-channels. **(A)** Here, all components are laid out on a surface for display: A glass slide (*top*) and coverslip (*bottom right*), both cleaned according to the protocol, and five narrow strips of double-sided tape, with backing-paper covering both sides (*bottom left*). When constructing the flow-channels, do not let the center of the cleaned coverslip touch any surfaces. **(B)** Remove backing-paper from one side of each strip of tape, then adhere them onto the coverslip, forming parallel channels. Press firmly on the tape strips to adhere them completely to the coverslip. Avoid trapping air pockets. **(C)** Remove backing-paper from the other sides of the tape strips. **(D)** Adhere the coverslip to the microscope slide, with long-axes perpendicular, such that the coverslip overhangs past the edges of the slide. Flip the flow-channel assembly over, so the coverslip is on the bottom. **(E)** Use a syringe to deposit vacuum grease between neighboring channels, to separate their openings and prevent unwanted mixing of fluids that will later be applied manually with a pipettor.

The simple double-open-ended flow-channels are easy to make, require no custom-made parts (no bespoke aluminum hose-barb fittings), and sufficient for observing or quantifying kinetochore assembly at the single molecule level by TIRFM. They can easily be made with multiple parallel channels, allowing several independent assembly reactions to be examined side-by-side on a single coverslip (Figure 2).

**FIGURE 3:**
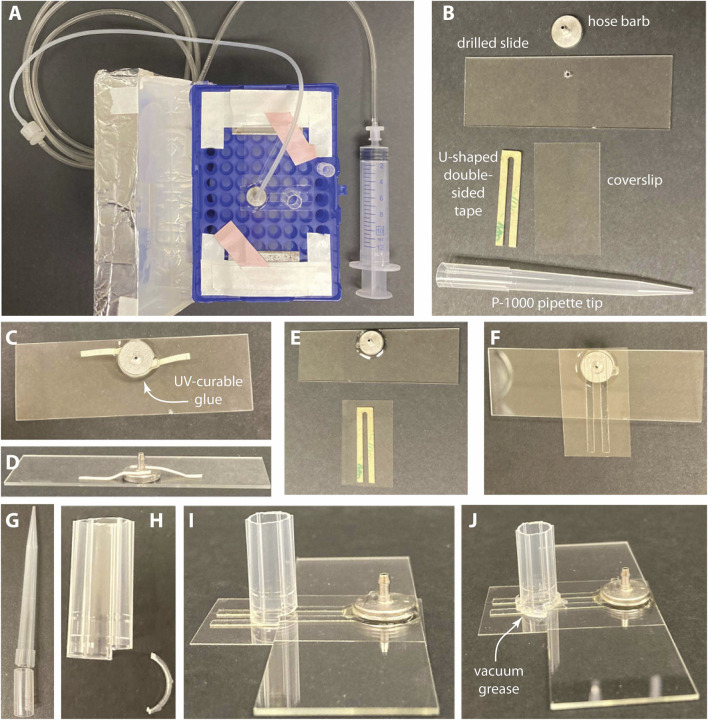
Constructing a pump-driven flow-channel. **(A)** Completed flow-channel stored in a modified pipette-tip box. The flow-channel rests on two razor blades to keep the coverslip from contacting the box. The syringe attaches to the hose barb via flexible tubing. A stack of two P-200 tips keeps the lid of the box from touching the top of the reservoir when closed. **(B)** Components of a pump-driven flow-channel. Both the drilled microscope slide and the coverslip should be cleaned according to the protocol before assembly of the channel. **(C–J)** Step-by-step construction procedure: **(C)** Temporarily tape the hose barb to the microscope slide such that the center of the hose barb aligns with the hole in the slide. Apply UV-curable glue to the base of the hose barb. Harden the glue with a UV-lamp or a gel transilluminator. **(D)** The temporary tape, shown here in a side-on view, should leave room for the glue to flow completely around the base of the hose barb. It is removed once the glue is cured. **(E)** Remove the backing paper from one side of the U-shaped double-sided tape and then press it firmly onto the coverslip. **(F)** Remove the backing paper from the second side of the tape. Align the coverslip and U-shaped tape with the slide, such that the bottom of the U is centered on the hole in the slide. Press firmly on the coverslip to adhere it to the slide. Turn the flow-channel over so the coverslip is underneath. **(G)** Create a reservoir from the base of a P-1000 pipette tip. First, sever the tip into two parts by cutting approximately 2 cm from the base. Discard the pointed end. **(H)** Next, cut a step into the reservoir to accommodate the microscope slide. Make two shallow cuts lengthwise into the base, approximately 1 mm deep, not quite halfway around the circumference. Remove and discard the C-shaped piece between these two cuts. **(I)** The stepped reservoir should fit onto the coverslip with a small section overhanging the microscope slide, as shown here. Press the reservoir onto the double-sided tape to keep it in place. **(J)** To make the reservoir water-tight, use a syringe to deposit vacuum grease around its base. Pay special attention to seal the area where the reservoir steps up over the microscope slide, and where it crosses the gap between the two arms of the double-sided tape.

1. Place a slide and a coverslip in a clean storage box, making sure that nothing touches the middle of the slide or coverslip.
2. Make thicker double-sided tape by sandwiching a layer of clear polyester film between 2 strips of 3M acrylic adhesive.
3. Cut a section of the thick tape just short of the length of the coverslip, then cut it into strips about 2 mm wide (Figure 2A).^8^
4. Remove the protective backing paper from one side of each tape strip and use them to make channels on the coverslip about 2 mm apart (Figure 2B). There is enough room to make up to four side-by-side channels using this method. Leave the other side of the tape covered with the protective backing paper.
5. Use the wide end of a clean pipette tip to press on the backing paper, firmly adhering the tape to the coverslip while avoiding or eliminating air pockets that can become trapped in the adhesive.
6. Remove the protective backing paper from the other side of the tape strips (Figure 2C). Orient the long-axis of the coverslip perpendicular to the microscope slide, so that the ends of the coverslip project outward past the edges of the slide (Figure 2D). Firmly press the coverslip down with the pipette tip to adhere the tape and coverslip onto the slide and eliminate air pockets that can become trapped in the adhesive. Against a dark background, air pockets should be obviously lighter in color than the surrounding tape.
7. Flip the entire channel assembly over, so the coverslip is on the bottom, with the open ends of the channels accessible on either side, where the coverslip projects outward beyond the slide edges. Use a syringe fitted with a pipette tip to deposit vacuum grease dams separating each channel opening to prevent mixing between the channels (Figure 2E).
8. Store the assembly in a humid box, keeping the coverslip from contacting any surface. We use a pipette tip box with razor blades taped on either side to elevate the slide (Figure 3B). The lid of the box is covered with aluminum foil to protect the assembly from light and a paper towel in the bottom of the box is soaked with water to maintain humidity.
9. Proceed to passivate the channels as described in part 2.4.4.

#### 2.4.3 Preparation of pump-driven flow-channels with reservoir

The pump-driven flow-channel is a modified chamber that we use specifically for our microtubule-capture and flip-flop assays. Instead of using a hand-held external vacuum line to manually aspirate solutions through channels with openings at both ends, we fit a syringe onto one end of a channel using a custom-made metal hose barb fitting, and we cover the other end of the channel with a relatively large fluid reservoir, cut from a pipette tip (Figure 3A). Then we use a syringe pump to precisely control the flow through the channel. The pump-driven channels are cleaned and passivated in the same way as the simpler double-open-ended channels, but they require a hole drilled through the microscope slide and the channels are constructed in a different way. The pump-driven flow-channels also usually need higher volumes of each reagent to fully exchange the volume inside the channel, reservoir, and hose barb.

1. Prepare the microscope slide by drilling a small (∼1 mm diameter) hole approximately 4–5 mm from the long edge (Figure 3B). Go slow, use water as cooling fluid, and try as much as possible not to chip or crack the glass around the hole. Rinse away any particulate debris left by the drill bit. It is best to prepare multiple slides at once all in a stack. This prevents chipping and cracking the glass. We use a drill press fitted with a fresh diamond-tipped drill bit and plenty of cooling fluid.
2. Clean the coverslips and slides as described in the slide cleaning section.
3. Take the drilled slide out of the mailer and place it in a clean storage box. Place the hose barb directly over the hole in the slide and use small strips of tape to hold it loosely in place (Figures 3C,D).^9^
4. Use a pipette tip to distribute UV-curable glue (Norland Blocking Adhesive 108) all the way around the base of the fitting. Check from below to make sure the glue has flowed completely underneath the fitting and that the holes in the hose barb and the slide still line up with each other.
5. Place the slides under UV light at 365 nm and allow the glue to cure until fully set, about 15 min.
6. Cut a piece of the thick double-sided tape to be slightly smaller than the length of the coverslip. Make a long U-shaped channel in the middle of the tape. The channel should be about 2–3 mm wide and about 70% the length of the tape.^10^ Leave the paper backing on one side of the tape (Figure 3E).
7. Use a pipette tip to firmly press the tape into the coverslip and remove any air.
8. Remove the paper backing and line the coverslip up so it is perpendicular to the microscope slide, and so the end of the U-shaped tape is directly behind the hole in the microscope slide (Figure 3F).^11^
9. To make the reservoir, cut an unfiltered P-1000 tip about 2 cm from the base (Figure 3G). At the base of the reservoir, use a razor to cut about 1 mm into the plastic at two points on a straight line less than halfway through the circle. Cut away the plastic between these cuts (Figure 3H), making room for the reservoir to slightly overhang the microscope slide.^12^
10. Press the reservoir into the tape overhanging the microscope slide (Figure 3I). Use vacuum grease to seal the base of the reservoir (Figure 3J). Be careful to completely seal the area where the reservoir steps up over the microscope slide, and where it crosses the gap between the two arms of the double-sided tape, so that the reservoir will not leak.
11. The flow-channel is ready for PEGylation at this point.
12. Modified pipette tip boxes can be used to store completed flow-channels (Figure 3A), so they are protected until use. One way to protect the reservoir is to stack two pipette tips and place them on the grid to prevent the lid from closing all the way. To minimize evaporation, maintain humidity in the box by placing a damp paper towel under the grid, in the bottom of the box.
13. Attach a syringe via tubing to the hose barb to pull liquid through the channel. Before attaching the syringe, fill it with water and avoid introducing any air bubbles into the tubing.
14. Proceed to passivate the flow-channel as described in part 2.4.4. To make sure the channel is fully passivated, continue to add PEG solution until a small bead of the solution appears at the opening of the hose barb. The pump-driven flow-channels are more likely to dry out and need additional solution at each end to minimize evaporation.

#### 2.4.4 Passivation of flow-channels with polyethylene glycol (PEG)

1. Prepare a 100 mM solution of sodium bicarbonate in MilliQ water and filter it with a 0.22 μm PES syringe tip filter.
2. Weigh 2–5 mg of PEG-biotin into an Eppendorf tube using a microbalance.^13^
3. Spin down the PEG-biotin and then add 100 µL of sodium bicarbonate solution for every mg of PEG-biotin.^14^ Vortex and centrifuge several times to ensure the PEG-biotin is fully dissolved.
4. Weigh 50–100 mg mPEG into an Eppendorf tube using a microbalance.
5. Spin down the mPEG and add 2 µL of PEG-Biotin solution from the previous step for every mg of mPEG.^15^ Vortex and centrifuge several times to ensure the mPEG is fully dissolved.
6. Manually pipette 20–40 µL of the mPEG/biotin-PEG mixture into each dry (empty) channel, filling the entire channel with no air bubbles.^16^ ^,^ ^17^
7. Put a wet paper towel in the base of the storage box, and cover with a lid to maintain high humidity. Leave the box overnight at room temperature.^18^ ^,^ ^19^
8. Both open-ended and pump-driven flow-channels are best if used the next day, but can be stored for up to a week at 4 °C with satisfactory results.

### 2.5 Assembly of kinetochores in flow-channel

On the day of the assay, gather the passivated, biotinylated flow-channel, biotinylated fluorescent centromeric DNA, whole cell yeast lysate with a fluorescently tagged kinetochore component protein, and fluorescent taxol-stabilized microtubules. The assay requires pulling a series of solutions through the channel and letting them incubate for specific amounts of time. Our simple double-open-ended flow channels rely on external suction to manually pull each solution through the channel (e.g., suction from a house vacuum line, or from a Drummond Scientific, Pipet-aid benchtop diaphragm pump). Our pump-driven flow channels use the attached syringe pump to pull each solution through and require higher volumes to account for the reservoir. After each incubation, wash with fresh 1xBRB80. In series, we incubate with avidin, then the biotinylated DNA, and then the yeast extract, which is spiked with salmon sperm DNA to prevent non-specific DNA binding proteins in the extract from competing with the kinetochore proteins for the centromeric DNA on the slide. Incubation times with the yeast extract depend on the concentration of the extract and the purpose of the experiment. Complete kinetochores take almost 90 min to assemble, but many of the inner kinetochore proteins arrive within 10 min. For a microtubule capture experiment, the full 90-min incubation in lysate is necessary, and it is good practice to use a yeast lysate with a fluorescently tagged outer kinetochore component, to monitor when the kinetochores are fully assembled and ready to bind microtubules. For an assembly assay only, without microtubule capture, image collection can start immediately after washout of the yeast lysate. For microtubule capture and flip-flop experiments, additional incubation with microtubules is required. A 10-min incubation with microtubules is usually enough, though if microtubule binding is low, increasing the duration might help. At each step, check that the desired density has been reached. If density is low, additional incubations with the same solution will usually increase the density.

1. Prepare for the assay by gathering and preparing the following reagents:
  5x BRB80 (400 mM PIPES pH 6.8, 5 mM MgCl_2_, 2.5 mM EGTA).
  1x BRB80 (dilute 5x BRB80 with MilliQ water).
  MilliQ water.
  1 mg/mL Avidin DN.
  10 mg/mL Bovine Serum Albumin.
  2 μg/mL salmon sperm DNA.
  5 mg/mL Kappa Casein in BRB80.
  Yeast lysate, one aliquot (prepared as described in section 2.1 above).
  PEG-passivated flow-channel (prepared as described in section 2.4 above).
2. Use 400 µL of 1xBRB80 to wash excess PEG from the channel. Add just enough volume to cover the input end of the channel and use a vacuum line or filter paper to pull the liquid through.^20^ Continuously apply BRB80 to one side of the channel until all of it has been pulled through. Be careful to avoid pulling air into the channel.
3. Add at least 50 µL of 0.1% (w/v) BSA in 1xBRB80 to the channel to block any areas of the coverslip that were poorly passivated by the PEG. Incubate for 5 min.
4. Wash the channel with 200 µL of 1xBRB80.
5. Add at least 50 µL of 0.33 mg/mL Avidin DN in 1xBRB80 and incubate for 5 min.
6. Wash the channel with 200 µL of 1xBRB80.
7. Dilute the DNA to between 50 and 800 pM in BRB80 depending on the desired density.^21^ Add at least 50 µL of diluted DNA to the channel and incubate for 5 min.
8. Wash the channel with 200 µL of 1xBRB80.
9. Check the density of DNA using the TIRF microscope.^22^ If it is too low, repeat the steps 7 and 8 with a higher DNA concentration.
10. Thaw an aliquot of yeast extract. For every 50 µL of extract, add 0.8 µL of 2 μg/mL salmon sperm DNA. Incubate on ice for 15 min.
11. Add 100 µL of yeast extract with salmon sperm DNA and incubate at room temperature for 90 min.^23^
12. Wash the lysate away with 200 µL of 1xBRB80.
13. At this point, the channel surface can be imaged to quantify colocalization between the DNA molecules and the labeled kinetochore subcomplexes. For microtubule binding assays continue to the next section, 2.6.

### 2.6 Capturing microtubules with assembled kinetochores

**FIGURE 4:**
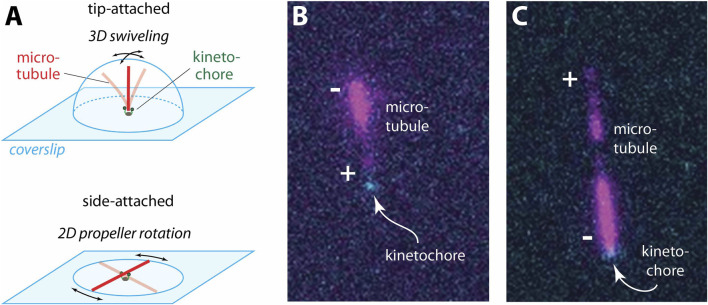
Determining kinetochore tip-attachment configuration with polarity-marked microtubules. **(A)** Tip-attached microtubules can be identified by the way they move. A tip-attached microtubule swivels in three dimensions, often pointing up away from the coverslip, where it is out of focus and outside the TIRF illumination. Conversely, a side-attached microtubule rotates around its kinetochore attachment like a propeller, remaining in focus. For clear viewing, a gentle flow of buffer can be applied using a syringe pump to keep the kinetochore-attached microtubules parallel to the coverslip and in the plane of focus. **(B)** Typical example where an individual, assembled kinetochore (*cyan*) has captured a polarity-marked microtubule (*magenta*) by its dimmer, plus end. **(C)** Rare example of a polarity-marked microtubule captured by its brighter, minus end.

1. Previously prepared microtubules can be diluted as needed. Some preparations cannot be diluted while others can be diluted up to 5x. Add κ-casein to a final concentration of 0.5 mg/mL. A minimum volume of 70 µL is needed.^24^
2. Place the syringe attached to the flow channel (section 2.4.3, step 13, above) into a controlled syringe pump. Set the pump to withdraw 0.05 mL (50 µL) of volume at a rate of 0.6 mL/min or less. Make sure to use a pipette to completely aspirate any additional BRB80 from the reservoir before adding the microtubules with kappa casein prepared in the previous step. Start the syringe pump to pull the microtubules through the channel. Watch to make sure that no air gets pulled through and be ready to stop the pump.
3. Incubate for 10–15 min.
4. Wash with ≥200 µL of 1x BRB80. Use the syringe pump to pull the solution through the channel at a gentle flow rate of 0.6 mL/min or less.
5. Add 200–400 µL of 1xBRB80 to the reservoir until it is about half full. Set the syringe pump to continuously oscillate with a flowrate of 0.3–0.6 mL/min,^25^ and a total volume per stroke of 0.2 mL. Watch the reservoir carefully for the first full cycle to make sure that it does not run dry or overflow.
6. Image the microtubules as they move with the buffer (Figure 4). For the polarity preference assay, at least one full reversal of flow is needed to determine the end attachment status of each microtubule. For the flip-flop assay, three full reversals of flow are recommended.

### 2.7 Image acquisition

**FIGURE 5:**
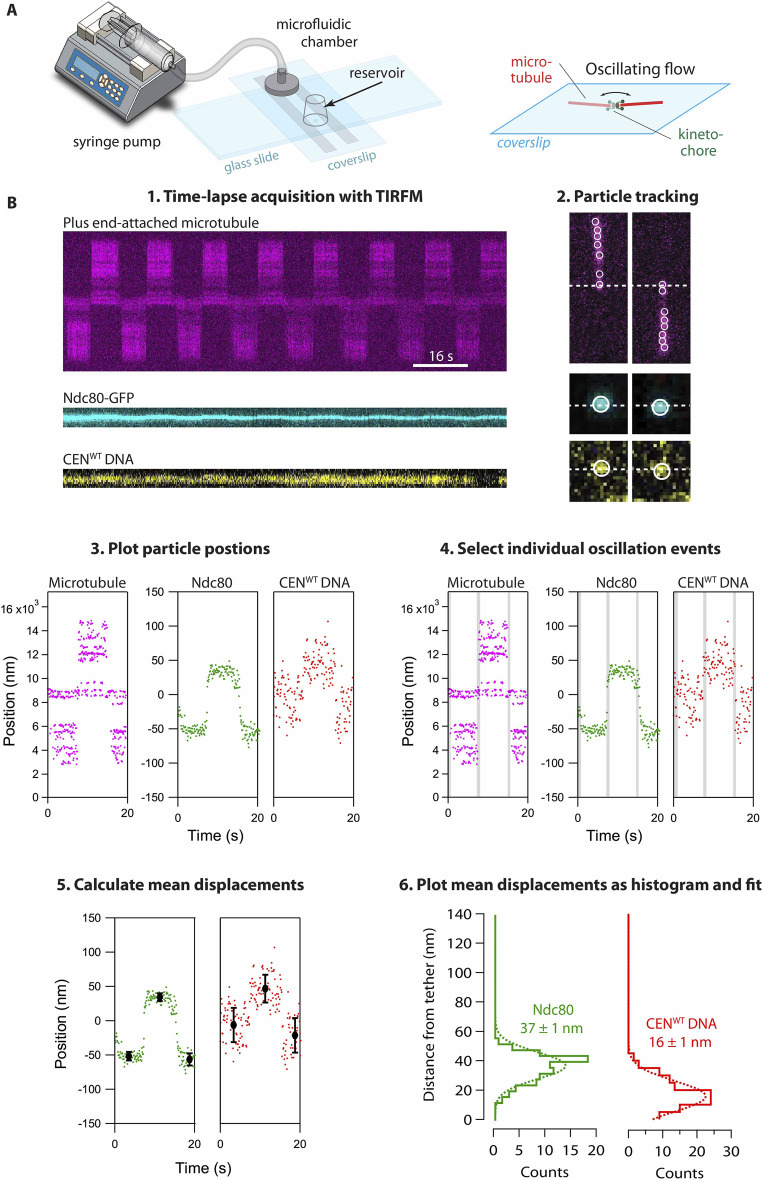
Measuring kinetochore subcomplex positions. **(A)** Kinetochore-captured microtubules are subjected to oscillating flow using a flow-channel attached to a syringe pump, prepared as described in Section 2.4.3 (see also, Figure 3), and used as described in Section 2.6. As the microtubule reorients with the oscillating flow of buffer, the attached kinetochore is reoriented with it, and displacements of labeled subcomplexes relative to the tether point, where the kinetochore is anchored on the coverslip, can be measured using TIRFM. **(B)** Steps for measuring kinetochore subunit displacements: 1. Kymographs show oscillations of the microtubule (*magenta*), kinetochore subcomplex (Ndc80, *cyan*), and centromeric DNA (*yellow*). 2. Particle trajectories are tracked in ImageJ using the MOSIACsuite 2D particle trajectory. 3. Positions of the individual tracked particles are plotted against time. 4. Intervals during microtubule reorientation are excluded (*grey shaded intervals*). 5. Mean displacements are calculated during each interval when the microtubule orientation was steady. 6. Histograms of mean displacements for oscillations from dozens of kinetochore subunits and DNAs with gaussian fits. The GFP tag on Ndc80-GFP is located 37 nm from the tether point (116 measurements across 19 kinetochore assemblies). The Atto565 dye on the centromeric DNA is located 16 nm from the tether point (74 measurements across 13 kinetochore assemblies).

Acquiring long videos with multiple flow oscillations requires minimizing exposure to the excitation lasers before acquiring images. It is advantageous to have the flow oscillating continuously while searching for kinetochore-captured microtubules. This allows tip-captured microtubules to be easily identified by their characteristic ‘flip-flop’ behavior (Figure 5A). Because the microtubules are bright and carry many fluorophores, they are more resistant to bleaching than the GFP-labeled kinetochore particles or the singly labeled DNAs, so it is best to search while only directly exciting the microtubule-specific fluorophore. This strategy minimizes the chances of photobleaching GFP-labeled kinetochore particles before acquisition begins and helps generate long movies with many oscillations (Figure 5B, *step 1*). Once suitable kinetochore-captured microtubule(s) are located, a quick spot-check is useful to ensure that the surface-assembled, GFP-labeled kinetochore and the corresponding DNA remain in focus. The optimal excitation power and exposure time will vary depending on the specifications of the TIRFM instrument and the goal of the experiment. For the instrument used here, the laser power at the sample was approximately 100 to 1,000 µW for each excitation source and the exposure time was ≥200 ms. Images were acquired until the GFP signal from the kinetochore was no longer detectable, the microtubule got stuck to the surface, or a satisfactory number of oscillations occurred. Movies captured during these experiments can last up to several minutes and typically include four to fifteen ‘flips’.

### 2.8 Data analysis

After image acquisition, individual TIFFs for each color channel are stacked in ImageJ. Particle tracking for the DNA, kinetochore, and microtubule are performed separately using the MOSAICsuite 2D particle tracker plugin for ImageJ (Sbalzarini and Koumoutsakos, 2005). For microtubule tracking, open the corresponding stacked TIFF in ImageJ and crop it around the microtubule to minimize erroneous particle tracking. Open the MOSAICsuite 2D particle tracker under *Plugins* and set the particle detection parameters so that multiple spots are selected along the length of the entire microtubule (Figure 5B, *step 2*). For the microtubules in the example data shown here, the following particle detection parameters were used: *Radius* = 3 or 5, *Cutoff* = 0.001, *Per/Abs* = 0.25 to 1.0. Since there is no need to track individual particle trajectories for the microtubule, the particle linking parameters are not critical. (For the microtubules in the example data shown here, the particle linking parameters were *Link Range* = 3, *Displacement* = 3, *Dynamics* = Brownian.) Once appropriate parameters have been found click *okay*. In the results window, select *Visualize All Trajectories* to check the accuracy of the particle tracking. There may be some erroneous particles that were tracked, but because of the large number of tracked particles, a few erroneous particles will not impact subsequent steps in the analysis. If the tracked particles are satisfactory, select *All Trajectories to Table* and save them for import into Igor Pro. The process for tracking the kinetochore and DNA markers are similar with two notable exceptions. Because the goal is to be able to track and quantify the displacement of individual kinetochores and DNA molecules, it is important that the particle tracking and linking parameters are set so there is only one continuous trajectory for each marker. For tracking GFP-labeled kinetochores, the particle linking parameters used here were: *Link Range* = 3, *Displacement* = 3, *Dynamics* = Brownian. Once satisfactory tracking is achieved, select *Visualize All Trajectories* in the *Results* window and select the trajectory of the particle of interest. Generate a table of particle positions by selecting *Selected Trajectory to Table* and save for import into Igor Pro.

Particle trajectories are individually loaded into Igor Pro and analyzed with custom scripts. The trajectories are plotted (Figure 5B, *step 3*) and intervals during which the microtubule orientation remained steady are manually selected (Figure 5B, *step 4*; the excluded intervals, during flow reversals when the microtubule was reorienting, are shaded gray in this figure). Mean positions and standard deviations are then calculated during each steady interval (Figure 5B, *step 5*). The position of the biotin-avidin tether point on the coverslip surface is inferred as the midpoint between mean positions before and after each flow reversal. To estimate displacements from the tether point, the pairwise difference in mean positions between consecutive steady intervals is divided by two. Distributions of mean displacement for many oscillations, recorded with many individual tip-attached kinetochores, are then plotted and fit with Gaussians (Figure 5B, *step 6*). Figure 5B, step 6 shows the distributions for 74 measurements of DNA displacement across 13 individual kinetochore assemblies, and for 116 measurements of Ndc80 displacement across 19 individual kinetochore assemblies. All custom software and algorithms used for the analysis are freely available (Larson et al., 2025).

### 2.9 Summary

The protocols detailed above allow direct examination of kinetochore assembly and microtubule capture at the single-molecule level. With super-resolution tracking, the arrangement of individual subunits within both tip-attached and side-attached kinetochores can be mapped at nanometer scale. The robustness of kinetochore assembly and microtubule capture enable consistent and repeatable measurements, and recently revealed that kinetochores adopt different architectures depending on the direction of external load relative to the microtubule’s structural polarity (Larson et al., 2025). Ideally, these types of measurements will be expanded to also include components of the spindle assembly checkpoint and the chromosomal passenger complex, which are both thought to sense the type of microtubule attachment and the amount or direction of force at the attachment point.

## 3 Reagents

### 3.1 Microtubule preparation

**Table:** 

Item	Catalog number	Vendor
N-Ethylmaleimide	040526.03	Fisher scientific
β-Mercaptoethanol	M3148-25 ML	Millipore-sigma
Tubulin protein (fluorescent HiLyte 647): porcine brain	TL670M	Cytoskeleton, Inc
DTT	D0632-5G	Millipore-sigma
GMPcPP	JBS-NU-405S	Jena biosciences
Dimethyl sulfoxide	BP231-100	Fisher scientific
Guanosine 5′-triphosphate sodium salt hydrate	G8877-1G	Milipore sigma
Paclitaxel	T7191-5 MG	Millipore sigma

### 3.2 Flow-channel preparation

**Table:** 

Item	Catalog number	Vendor
Cover slip	12–544-C	Fisher scientific
Gold seal plain microscope slides (75 mm × 25 mm) (pack of 144; case of 25 pack)	12-518-100B	Fisher scientific
5-Slide mailer, end-opening, natural color	HS15986	Fisher scientific
Cole parmer MICRO-90 cleaning solution	NC0248628	Sigma
Ethanol, anhydrous, denatured, BAKER ANALYZED* reagent (ACS grade)	JT9401-06	VWR
Potassium hydroxide (pellets/Certified ACS)	P250-1	Fisher scientific
Acetone, HPLC grade	A949-1	Fisher scientific
Vectabond	SP-1800	Vector labs
NITROGEN 99.999% UHP T, ultra high purity grade	NI 5.0UH-T	Praxair
mPEG-SVA, Methoxy poly(ethylene glycol) succinimidyl valerate, MW 5000	MPEG-SVA5000-1g	Laysan bio
Biotin-PEG-SVA, MW 5000	Biotin-PEG-SVA, MW 5000-100 mg	Laysan bio
Acrylic adhesive	300LSE	3M
Mosture-resistant polyester film	8567K12	McMaster-Carr
Norland blocking adhesive 108	C006968-6	Norland

### 3.3 Kinetochore assembly and microtubule capture

**Table:** 

Item	Catalog number	Vendor
Albumin, bovine serum, Fraction V, Fatty acid-free, nuclease- and protease-free	126609-10 GM	CalBioChem
Avidin DN	A-3100	Vector labs
Salmon sperm DNA	AM9680	Fisher scientific
Glucose oxidase	G2133-50KU	Millipore-sigma
Catalase	C315-110 MG	Millipore-sigma
Kappa-casein	C0406-1G	Millipore-sigma

## Data Availability

The original contributions presented in the study are included in the article/supplementary material, further inquiries can be directed to the corresponding author.

## Footnotes

1 Make 100 mL of lysis buffer and store at 4 °C, adding DTT and protease inhibitors just before use

2 The volume of lysis buffer added can be calculated using either the pellet weight or the concentration of cells (OD and volume at harvest).

3 Cells can be stored at −80 °C at this point

4 For single-molecule experiments, our aliquots are 100 µL.

5 Our slide mailers have 5 slots, so we usually prepare two slides at once. Prepared flow-channels will keep for about a week at 4 °C

6 You will only need one coverslip for each flow-channel, but it is best to prepare an extra one in case of breakage

7 For pump-driven flow-channels, skip ahead to follow part 2.4.3 below. For simple double-open-ended flow-channels, proceed to part 2.4.2.

8 A laser cutter can be used to pre-cut channels into a piece of tape sized for the coverslip. This has the added benefit of making the channels exactly the same volume with parallel sides, increasing repeatability.

9 It is important that there is space all around the hose barb where the glue can flow. Avoid pressing the tape directly into the glass coverslip from the hose barb–pull it out to leave room for the glue.

10 Rather than cutting a U-shaped piece of tape, a pair of parallel strips can instead be used, as in our simpler open-ended channels. In this case, you will need to dam the open channel-end with vacuum grease later.

11 The channel should go from just behind the hole in the microscope slide all the way to the opposite edge of the slide.

12 Try to minimize overhang, as you will need to fill any holes with vacuum grease.

13 We portion out our mPEG and PEG-biotin as soon as it is received and write the mass to the nearest 0.1 mg on the tube. This reduces workload on preparation day, since dried PEG can be quite static-prone and difficult to weigh. Store the weighed portions of dry PEG at −20 °C with desiccant.

14 For example, if the PEG-biotin aliquot is 2.5 mg, add 250 µL of sodium bicarbonate solution.

15 The weight of the mPEG solution determines the final volume of PEG solution you will make. For large channels, or when working with many channels simultaneously, you will need more mPEG. For example, a pump-driven flow-channel may need 50 µL to fill the channel, reservoir, and hose barb. This would require an mPEG aliquot of at least 25 mg to make enough solution.

16 Try not to touch the slide or coverslip with the pipette tip, because pressure on the coverslip can cause it to delaminate from the tape, allowing liquid to flow between the channels.

17 Add an extra drop of solution at each end of each channel to prevent evaporation inside the channel.

18 For the pump-driven channels, incubation at 4 °C overnight, rather than at room temperature, can reduce evaporation.

19 The channel can be used after 3–4 h if you are in a hurry, but we find that overnight incubation gives better results.

20 It is easiest to let the BRB80 sit on the PEG for at least a minute before using the vacuum aspirator to wash it through the channel. The PEG solution can initially be quite viscous, especially if you have recently removed it from the fridge. Allowing the BRB80 to sit allows the PEG to dissolve and warm up, reducing its viscosity and facilitating its washout.

21 For colocalization experiments it is better to have lower DNA density, while some microtubule capture experiments benefit from higher density. It is wise to troubleshoot what density works best for your specific use.

22 For a colocalization experiment, the density should be low enough that there are very few overlapping DNA molecules. The number of molecules depends on the of size of the field of view for your particular TIRFM. For the experiments described here, there were approximately 200–300 DNAs in a circular field of view with a 55 µm diameter. For polarity preference and kinetochore architecture experiments, the DNA can be more dense, but it may take some troubleshooting to determine the ideal concentration, since the optimum depends on other factors like the specific lysate preparation being used.

23 The duration of this incubation may be different depending on the desired outcome for the experiment. Inner kinetochore components are assembled relatively quickly, within a few min, while outer kinetochore components may take the entire 90 min to assemble. The location of the GFP tag can also impact the time to assemble certain kinetochore components. For a lysate concentration of 40–80 mg/mL total protein, you can expect a relatively large proportion of fully assembled, active kinetochores after approximately 90 min. Note that if you are using a pump-driven flow-channel, it is imperative that you remove excess BRB80 from the reservoir prior to adding the lysate to avoid dilution of the lysate. Less concentrated lysate has much lower assembly efficiency.

24 Keep all of your reagents between 25 °C and 37 °C when working with microtubules to prevent depolymerization due to cold shock. This is especially important with polarity-marked microtubules.

25 The optimal flow rate depends on the rate of fluorophore bleaching and the stability of your kinetochore-microtubule attachments. For weaker attachments, or if you are seeing a lot of free microtubules, decrease the flowrate to reduce viscous drag forces on the kinetochore-microtubule interface, which can sometimes be strong enough to detach microtubules from surface-tethered kinetochores. If your fluorophores are bleaching before you can image at least 2 reversals of direction, either increase the flow rate or decrease the total flow volume to increase the frequency of reversals. For data analysis, you need to witness how the microtubules respond to the flow reversing direction.
